# Battle of the axes: simulation-based assessment of fine needle aspiration biopsies for thyroid nodules

**DOI:** 10.1186/s40463-022-00587-5

**Published:** 2022-08-19

**Authors:** Shireen Samargandy, Justine Philteos, Mirko Manojlovic Kolarski, Jason Xu, Eric Monteiro, Allan Vescan

**Affiliations:** Department of Otolaryngology – Head and Neck Surgery, Mount Sinai Hospital, University of Toronto, 600 University Ave., Toronto, ON M5G 1X5 Canada

## Abstract

**Importance:**

Ultrasound-guided fine-needle aspiration biopsies (UGFNA) play a crucial role in the diagnosis of thyroid nodules. There are two techniques for performing an UGFNA: short-axis technique and long-axis technique. There is sparsity in the literature regarding the differences between these two techniques.

**Objective:**

To compare the efficiency between long-axis and short-axis thyroid UGFNA techniques in trainees. Our secondary outcomes were to define the comfort level and learning curves of trainees.

**Design:**

A longitudinal prospective cohort study, completed from December 2018 to November 2019, using the *Blue Phantom Thyroid Model©* for UGFNA. Face and construct validity of the model were verified. Residents completed UGFNA on an assigned nodule using both long-axis and short-axis techniques, the order of which was sequentially allocated. The rate and time to successful biopsy were obtained for both techniques. Biopsy attempts were repeated to establish learning curves.

**Setting:**

Single-center study.

**Participants:**

Fourteen Otolaryngology—Head & Neck Surgery residents at the University of Toronto.

**Main outcome measure:**

Biopsy success and efficiency for novice learners completing UGFNA on a simulated thyroid model using long-axis and short-axis techniques.

**Results:**

A trend towards higher odds of successful biopsy using the long-axis technique with no difference in procedure duration was observed (OR = 2.2, *p* = 0.095, CI = 0.87–5.39). Learning curve graphs appeared heterogenous according to trainee level. Trainees found the long-axis technique easier to perform (10/14, 71%), and the simulator valuable for learning (12/14, 86%).

**Conclusion:**

Thyroid UGFNA using the long-axis technique may have an increased success rate and is generally favored by trainees for being easier to perform. Thyroid simulators have the potential to increase learner comfort and efficiency with UGFNA.

**Graphical Abstract:**

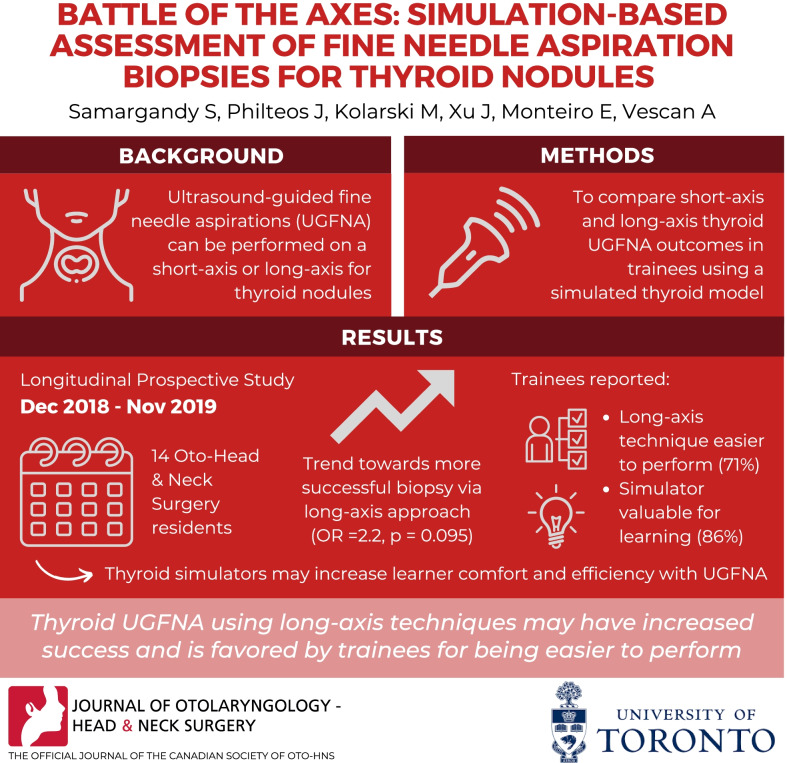

**Supplementary Information:**

The online version contains supplementary material available at 10.1186/s40463-022-00587-5.

## Introduction

Thyroid nodules are highly prevalent and can be present in more than 50% of the general population, as suggested by autopsy data [1]. Suspicious thyroid nodules are often investigated with an ultrasound-guided fine-needle aspiration (UGFNA) [2] biopsy. In the work-up of suspicious nodules, it is important to obtain an accurate UGFNA biopsy. It has been estimated that 11% of UGFNA biopsies are non-diagnostic due to insufficient sampling [3, 4]. Non-diagnostic results necessitate repeat biopsies, delays treatment decisions, and result in increased anxiety among patients and frustration among clinicians [5].

There are two methods of performing an UGFNA: long-axis and short-axis techniques. In the long-axis (in-plane) technique, the needle is inserted obliquely along a parallel path to the image plane allowing the needle tip and shaft to be visualized throughout the entirety of the procedure. In contrast, in the short-axis (out-of-plane) technique the needle insertion point is perpendicular to the ultrasound transducer with visualization of the needle tip as a bright echogenic focus in the center of the nodule being biopsied.

Thyroid fine needle aspiration (FNA) is a valuable skill for trainees in Otolaryngology–Head & Neck Surgery (OHNS), Endocrine Surgery, Endocrinology, and Interventional Radiology. Simulation can facilitate trainee acquisition of this skill. The goal of medical simulation is to replicate patient care scenarios in a realistic environment encouraging learning through trial and error [6, 7]. Training models have been used in teaching many procedural skills, including PICC line insertion, peripheral intravenous access, transvaginal ultrasound examinations, and endobronchial ultrasounds [8–14]. Our study compared the differences in biopsy success amongst OHNS trainees using long-axis and short-axis techniques on a thyroid UGFNA simulation training model. In addition, we examined the learning curves associated with the utilization of these techniques.

## Materials and methods

### Study design

We conducted a longitudinal prospective cohort study. Our primary outcome was to delineate the differences in accuracy and efficiency between long-axis and short-axis thyroid UGFNA techniques in trainees. Our secondary outcomes were to define the comfort level and learning curves of trainees utilizing both these techniques. We also assessed the validity of the *Blue Phantom Thyroid Model* in UGFNA. The study participants included PGY1-4 residents from the Department of OHNS at the University of Toronto in Toronto, Canada. Fourteen out of seventeen eligible residents joined the study. We received ethics approval from the Mount Sinai Hospital Research Ethics Board and obtained consent from all participants.

### Pre-simulation and simulator

We first administered pre-test questionnaires to the participants to assess demographics and previous UGFNA experience (Appendix 1). The participants then watched three mandatory short instructional videos outlining how to use the ultrasound machine assigned to the study, as well as perform a long-axis and short-axis UGFNA of a thyroid nodule **(**Additional files 2, 3, 4).

**Fig. 1:**
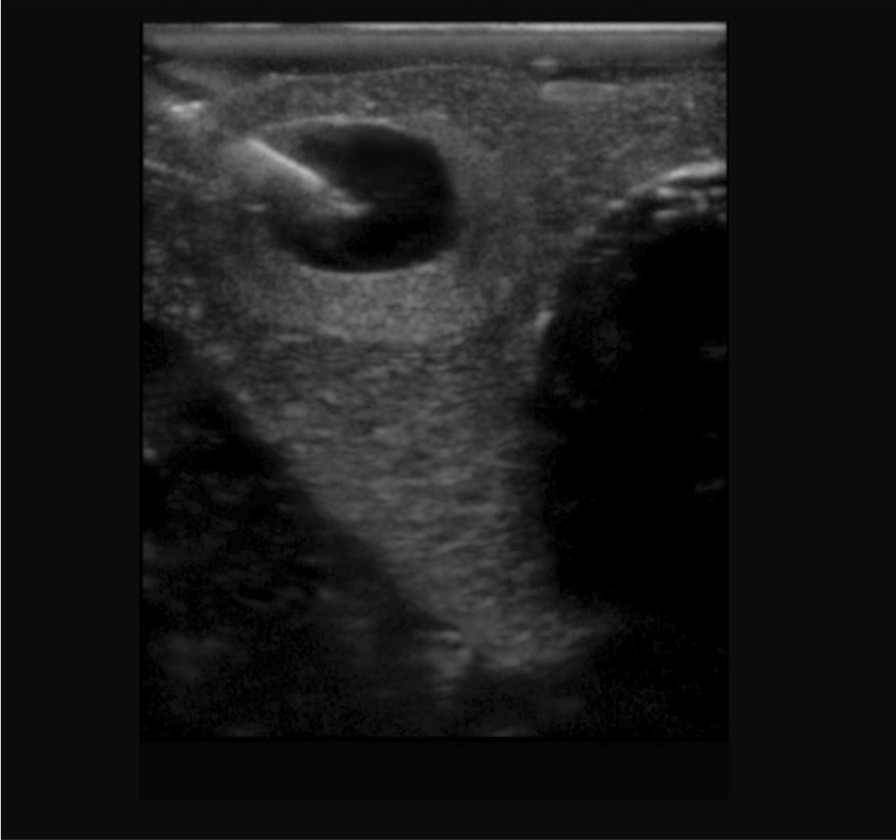
Blue Phantom Thyroid Simulator Model© Thyroid Nodule Example. This figure shows an image of the right inferior nodule in the simulated thyroid gland with a needle inserted in long-axis for an aspiration attempt [26]

The *Blue Phantom Thyroid Model ©* was the thyroid simulator used for this study. It is a simulated neck containing a thyroid gland with some of the adjacent anatomical structures made from a patented, ultrasound-compatible material. We sequentially assigned the participants one of four nodules available on the model—superior right, superior left, inferior right or inferior left nodule. The nodules had different sonographic appearances: hypoechoic, isoechoic, anechoic with an echogenic rim, and hyperechoic with a hypoechoic rim**.** A sonographic example of a thyroid nodule is provided in Fig. 1.

### FNA evaluation and definitions

We instructed the trainees to use both long-axis technique or short-axis technique to biopsy their assigned nodule using a 25-gauge needle under observed, timed conditions. We defined an FNA “trial” as the visit in which the resident participated in the study. An FNA “attempt” was used to describe the FNA procedure, starting from the moment the needle enters the simulated skin. An FNA “re-attempt” was the event in which more than 75% of the needle shaft was withdrawn or removed altogether from the simulator and reinserted. We considered a biopsy successful when the needle-tip was identified within the assigned nodule. Time-to-successful-biopsy was defined as the time from needle insertion in the model and until the evaluator visualized the needle-tip within the nodule. Two of the authors (S.S and M.K) with experience in performing UGFNA using both techniques were involved in evaluating each FNA trial. They strictly observed and did not provide any feedback or assistance to study participants during the FNA trial.

Time-to-successful-biopsy and number of attempts to reach success for each FNA attempt were recorded for each FNA trial. The participant was then prompted to perform the biopsy using the alternate technique and was evaluated in a similar fashion. The evaluator would then rank the trainee on a global proficiency ordinal scale from a score of 1 (not proficient) to a score of 5 (very proficient) on the use of the ultrasound probe, needle handling, time and motion, flow of procedure, and overall FNA performance. The trainee’s performance was subsequently compared to expert-level proficiency (described in more detail in the construct validity section). Visits were repeated, up to 8 times, to assess the learning curves with both techniques. Trainees had the option to stop if they reached expert-level proficiency.

### Comfort levels

Participants were asked to identify which technique was easier to learn and perform, how many practice attempts were necessary to achieve proficiency on the model, and to identify the areas of most benefit from participating in the study. Furthermore, using a 5-point Likert Scale, subjective performance of UGFNA was assessed.

### Learning curves

Learning curves outlining the time-to-successful-biopsy using both techniques were explored visually using restricted cubic splines mixed effects models.

### Face validity and construct validity

Face validity was assessed to ensure the appropriateness of the selected simulator. Following participation in the study, each participant received a post-test questionnaire delineating the face validity of the thyroid FNA simulation (Appendix 2). Construct validity was determined by comparing resident learner and attending staff performance (Additional file 1). Residents were deemed to reach expert-level proficiency if they, at any trial, successfully completed the FNA within 20 s; corresponding to the 90th percentile of staff-level proficiency.

### Statistical analysis

Statistical analysis was performed using R version 3.6. The geepack package (version 1.2.1) was utilized for the generalized estimating equations model and the survival package (version 2.44.1) was used for the Cox proportional hazards model. A test was considered statistically significant when the *p*-value was < 0.05. Continuous variables are presented as a mean ± SD. The lme4 package (version 1.1.21) was used for learning curve modeling.

## Results

Participants partook in this study from December 2018 to November 2019. Study subjects’ characteristics are outlined in Table 1. A majority of participants were PGY1 or PGY2 residents (64%), male (86%) and right-hand dominant (86%). The mean age was 28 ± 2.1 years. More than half (57.1%) had previously used a simulation device in medical learning. Each participant completed, on average, 4.2 ± 2.1 FNA trials over 3.5 ± 1.8 months. The average interval between trials was 3.5 ± 3.4 weeks. One participant withdrew after only one visit due to scheduling conflicts. The limited sample size precluded covariate analyses.

**Table 1:** Demographics of study participants

Participant number	Age	Gender	Handedness	PGY level*	Previous experience with any medical simulator
1	27	Male	Right	3	No
2	26	Male	Right	1	Yes
3	29	Male	Left	1	Yes
4	28	Female	Right	4	Yes
5	27	Male	Right	1	Yes
6	27	Male	Right	4	Yes
7	31	Male	Right	2	Yes
8	27	Male	Right	2	No
9	28	Male	Right	2	No
10	27	Male	Right	2	No
11	28	Male	Left	3	Yes
12	34	Male	Right	4	Yes
13	25	Female	Right	1	No
14	28	Male	Right	1	No

*PGY post-graduate year

### FNA performance

Eleven participants completed a successful FNA on their first trial using both the long-axis and the short-axis techniques (79%). The remaining 3 participants were successful by their second trial using both techniques. Across all trials, 83.9% of biopsy attempts were successful irrespective of the UGFNA technique used (99/118). Overall, 53/59 (89.8%) of the long-axis attempts were successful compared with 46/57 (80.7%) of short-axis attempts. The long-axis technique yielded higher odds of successful biopsy (Odds Ratio = 2.1669); however, the difference did not reach statistical significance (95% CI: 0.873–5.386).

Two out of fourteen participants (14%) were unsuccessful in initial UGFNA attempt. If a participant was unsuccessful during the initial attempt, they required an average of 2.7 ± 1.3 re-attempts before achieving a successful UGFNA.

**Fig. 2:**
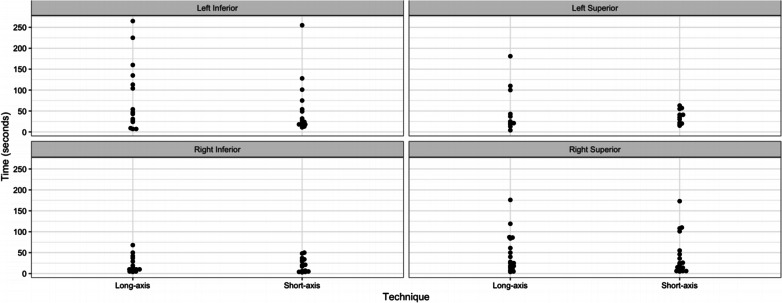
Time-to-biopsy-completion by technique for each tested nodule. This figure summarizes the time-to-biopsy-completion in seconds for each resident for all trials for a given nodule using both long-axis and short-axis techniques. Out of the 14 subjects, 8 eventually reached staff-level proficiency on at least one attempt. In these 8 subjects, the mean trials required to reach staff-level proficiency was 3.5

The average time taken to complete an FNA using the short-axis technique was 41 s (range; 3–255 s); whereas, the average time for the long-axis technique was 52 s (range: 4–265 s). For each nodule, Fig. 2 displays the total time-to-biopsy-completion per technique. The difference in the time taken to perform a biopsy between the two techniques was explored using a Cox proportional hazards model. There was no statistically observed difference in the time-to-successful-biopsy completion between short and long-axis techniques (Hazard Ratio = 0.94, *p* = 0.74).

The global proficiency scale was compared for each participant’s initial and final trial (Table 2). On the initial attempt, procedure flow and needle handling were the highest scoring skills (2.5 ± 1.4 and 2.5 ± 1.5, respectively). The skills that showed the most considerable improvement were overall FNA performance (∆ = 1.50 ± 0.49) and economy of movement (∆ = 1.50 ± 0.50).

**Table 2:** Evaluation scores for global proficiency scale

Skill analyzed	First trial proficiency*	Final trial proficiency*	Change in proficiency*^†^
Correct use of the ultrasound probe	2.21 (1.19)	3.50 (1.65)	1.29 (0.54)
Appropriate needle handling	2.50 (1.51)	3.71 (1.49)	1.21 (0.57)
Economy of Movement	2.36 (1.45)	3.86 (1.17)	1.50 (0.50)
Procedure Flow	2.50 (1.40)	3.93 (1.21)	1.43 (0.50)
Overall FNA Performance	2.43 (1.34)	3.93 (1.27)	1.50 (0.49)

*FNA* fine needle aspiration

*Data is presented as mean (± SD)

^†^Change in proficiency was calculated as an independent samples t-test for the difference between the mean of “Final Trial Proficiency” and the mean of “First Trial Proficiency.”

### Comfort levels

The majority of respondents found the long-axis technique easier to perform (10/14, 71.4%), and found it easier/no different to learn (9/14, 64%). In terms of subjective improvement for short-axis and long-axis FNA, the majority of participants believed they improved “somewhat” or “very much” in this skill (13/14, 92.8%).

### Learning curves

**Fig. 3:**
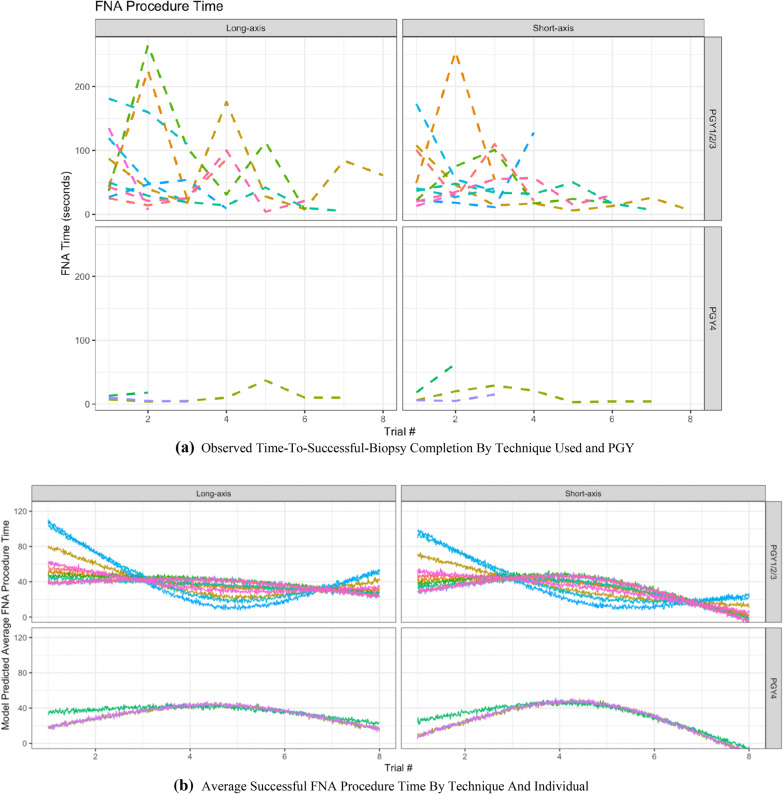
**a** Observed Time-To-Successful-Biopsy Completion By Technique Used and PGY Year. FNA: Fine needle aspiration. This figure is a visual representation of the learning curves for time-to-successful-biopsy completion for each resident in each trial, using both the short-axis and long-axis techniques. The upper graphic, is a representation of the data points for the junior residents in their PGY1-3 years; whereas, the bottom schematic visually represents data from the senior residents in their PGY4 year. The increased data variability in novice learners (PGY1-3) is noted. **b** Average Successful FNA Procedure Time By Technique And Individual. FNA: Fine needle aspiration. This figure is a cubic spline model of time to FNA completion in seconds over multiple attempts. It is a visual representation of the average learning curves for time-to-successful-biopsy across multiple attempts for each resident in each trial, using both the short-axis and long-axis techniques. The upper graphic, is a representation of the data points for residents in their PGY1-3 years; whereas, the bottom schematic visually represents data from residents in their PGY4 year. Again, The increased data variability in novice learners (PGY1-3) is noted

Learning curves outlining the time-to-successful-biopsy using both techniques were explored visually using restricted cubic splines mixed effects models as shown in Fig. 3a. Furthermore, the time-to-successful-biopsy completion was extrapolated across multiple attempts in Fig. 3b, which depicts the predicted FNA procedure time if all participants were to complete 8 trials. The learning curves show wide variability in time-to-successful-biopsy with initial attempts and a trend to faster completion with successive biopsies. Furthermore, 57% residents, 8/14, successfully reached staff-level proficiency on at least one trial– and required an average of 3.5 FNA attempts to reach this cut-off.

## Discussion

Despite the ubiquitous use of thyroid UGFNAs, to date there has been a paucity of literature comparing the short-axis and long-axis techniques. Proponents of the long-axis technique argue that the improved visualization of the entire needle track deems it easier to perform an UGFNA successfully; whereas, those in support of the short-axis technique note that it requires less hand–eye coordination and less challenging needle angulation [15, 16]. Both techniques provide comparable adequacy of cytologic samples for superficial nodules [17]. This study is the first to compare trainee performance in thyroid UGFNA and provide a visual analysis for the learning curves; furthermore, it demonstrates the possible utility of simulators in thyroid UGFNA training.

### FNA performance by technique

This study highlights a high first-pass successful biopsy attempt rate; which outlines that, to begin with, successful UGFNA is technically possible for the majority of participants. The long-axis technique resulted in a higher overall success rate compared to short-axis (89.8% vs 80.7%, respectively), however this did not meet statistical significance. A trend towards higher odds of successful biopsy using the long-axis technique with no difference in procedure duration was observed (OR = 2.2, *p* = 0.095). This may suggest improved success with this technique**,** but could not be confirmed with the available data. The ability to visualize the entire needle is a potential explanation for the improved biopsy success with the long-axis technique.

Of note, two of the FNA trials using the short-axis technique had an undetermined outcome due to the difficulty in visualizing the needle tip clearly during evaluation. This emphasizes a known shortcoming in this technique, as the suboptimal visualization makes it challenging to distinguish if a nodule was pass-pointed or missed. Kandil et al. compared outcomes of thyroid UGFNA using both techniques in patients [17]. They reported an increased diagnostic accuracy (95%) of the UGFNAs performed with the long-axis technique particularly in nodules deeper than 3 cm compared to short-axis (*p* = 0.01). Lesions superficial to 3 cm had comparable yields using both techniques. Studies comparing long-axis and short-axis techniques for peripheral venous access in a simulated setting similarly concluded that the long-axis technique was preferred due to the superior visualization of the entire catheter length, preventing the complication of posterior wall punctures [18, 19].

If a participant was initially unsuccessful in their first attempt, our study found that they required an average of 2.7 ± 1.3 re-attempts to achieve success. It is preferable to minimize the number of biopsy attempts to minimize procedure time and potential risk to surrounding structures. In a systematic review investigating FNA complications, Polyoz et al. reported an overall low risk of complications post-FNA; however, outlined complications including: infection, hematoma, recurrent laryngeal nerve palsy, tracheal puncture, and needle track seeding [20].

### Comfort levels

In our post-study survey, participants reflected on their improvement and comfort with thyroid UGFNA biopsies. The majority of the trainees (64%) felt that the long-axis technique was easier/no different to learn, and 71% reported that the long-axis was easier to perform. The post survey analysis suggests that although the long-axis may appear slightly more challenging at the beginning, it becomes the easier technique to use with successive attempts. This is in keeping with previous studies that have demonstrated positive effects of simulator training on resident comfort in performing thyroid UGFNA. Davis et al. assessed residents’ comfort levels in performing thyroid FNAs among 12 senior general surgery residents using a *Blue Phantom Ultrasound Central Line Training model©* with an embedded olive to simulate a nodule. At baseline, 62% felt “not comfortable” with the procedure; however, after practicing the aspiration technique, this was reduced to 0%. This further demonstrates that residents’ comfort in performing thyroid FNA is positively influenced through training on thyroid simulators [21].

### Learning curves

Learning curves were explored visually to gain further insight into UGFNA performance. We noted an increased variability in the performance of novice learners (PGY1-3) when compared to participants in their PGY4 year (Fig. 3a). The degree of unpredictability in PGY 1–3 decreases as trial number increases, suggesting an improvement in their UGFNA and skill acquisition. There was a positive learning trend for novice learners as repeated trials of UGFNAs led to improved speed of biopsy completion. In contrast, the more experienced PGY4 residents, who have performed numerous UGFNA throughout their residency training, had plateauing learning curves irrespective of the FNA trial number. The increased efficiency with repetition supports the use of thyroid simulators to provide an opportunity for additional practice with completing thyroid UGFNAs for residents early in their training.

### Simulation model: construct and face validity

Previous literature has also explored and demonstrated the benefit of ultrasound simulation, including *Blue Phantom Models ©,* in medical education [9, 21–25]. Construct validity is an important step in simulator evaluation and learning curve analysis, the model used in this study successfully differentiated between novice learners and experts (Additional file 1). The model had adequate face validity as the majority of residents responded that the simulator was an accurate representation of a thyroid biopsy experience (Additional file 1).

### Limitations

This study has important limitations that restrict the ability to draw broad conclusions based on its findings including limited sample size. Future studies to validate those findings in a larger group of trainees with a retention of skill analysis are needed. Furthermore, to accommodate the surgical residents’ schedules, the time intervals between study trials were variable. The simulated nature of our study design also limits generalization to clinical settings. Studies assessing predictive validity of the model can address that. Future studies will be aimed at correlating simulation findings with clinical performance.

There were drawbacks specific to the simulation model that are relevant. The simulated thyroid model left track marks from previous FNA attempts with continued use. This meant that participants had a visualized needle track to follow, which was not reflective of clinical practice. The authors are currently in the process of creating a more cost-effective, novel thyroid model to address this flaw.

## Conclusion

There is a dearth in the literature regarding the differences between employing the short-axis and long-axis techniques in UGFNA of the thyroid. Our results indicate that there may be an increased success rate with UGFNA using long-axis techniques, although statistical significance was not reached. The majority of trainees felt that the long-axis technique was easier to perform and learn. As such, the authors advocate for teaching both techniques starting with the long-axis technique to ensure proper visualization of the needle for novice learners. This study also shows the ability of a medical simulator to augment traditional training in obtaining UGFNA from the thyroid gland and calls for the improvement of simulators used in surgical training. Future studies with larger sample sizes are needed to determine predictive validity.

### Supplementary Information

**Additional file 1**. Construct validity of the thyroid fine needle aspiration simulator.

**Additional file 2**.** Video 1**: Ultrasound Knobology

**Additional file 3**.** Video 2**: Long-Axis Fine Needle Aspiration Biopsy

**Additional file 4**.** Video 3**: Short-Axis Fine Needle Aspiration Biopsy

## Data Availability

All data generated or analyzed during this study are included in this published article.

## Appendix 2: Face validity questionnaire

With regards to the simulated neck model used in this study, on a scale from 1 (*Strongly disagree)* to 5 (*Strongly agree)*, please indicate your opinion on the following statements:

## Abbreviations

UGFNA: Ultrasound-guided fine needle aspiration biopsy
FNA: Fine needle aspiration biopsy
OR: Odds ratio
CI: Confidence intervals
s: Seconds
PGY: Postgraduate year
