# Supplementary material for: Battle of the axes: simulation-based assessment of fine needle aspiration biopsies for thyroid nodules
Source: J Otolaryngol Head Neck Surg. 2022 Aug 19;51:32. doi: 10.1186/s40463-022-00587-5 (PMC9392298; doi:10.1186/s40463-022-00587-5)
Supplement: Supplementary file 1 — Additional file 1. Construct validity of the thyroid fine needle aspiration simulator. [file 40463_2022_587_MOESM1_ESM.docx]

**SUPPLEMENTARY MATERIALS**

**Supplementary Material 1**: Construct Validity of the Thyroid Fine Needle Aspiration Simulator

Nine Otolaryngology–Head & Neck Surgery attendings completed an UGFNA using both techniques on all four nodules in conditions similar to the trainees to establish expert-level thresholds. Staff participants were successful in 100% of their UGFNA trials regardless of the technique used; whereas, resident participants were only successful in 83.9% of their initial FNA attempts. Out of the 14 residents, 8 eventually reached staff-level proficiency, successfully completion of an FNA task in 8 seconds or less, on at least one attempt. Across all trials, regardless of nodule biopsied and technique used, the average time-to-successful-biopsy was 15.1 s ± 5.9 s among experts, and 45.3 s ± 9.3 s among the resident participants (independent-samples t-test = 21.9, *p* < 0.0001). Across all nodules and trials, 38.7% (12/31) of trials were successfully completed in 20 s (90^th^ percentile); whereas, 25.8% (8/31) were successfully completed in 8 s (50^th^ percentile).

**Supplementary Material 2**: Face Validity of the Thyroid Fine Needle Aspiration Simulator

| **Question** | **Answer** | **n (%)** |
| --- | --- | --- |
| **Were the model’s thyroid nodules realistic?** | Strongly Disagree | 0 (0.0) |
|  | Disagree | 1 (7.1) |
|  | Neutral | 3 (21.4) |
|  | Agree | 7 (50.0) |
|  | Strongly Agree | 3 (21.4) |
| **Was the simulator neck realistic?** | Strongly Disagree | 0 (0.0) |
|  | Disagree | 1 (7.1) |
|  | Neutral | 5 (35.7) |
|  | Agree | 4 (28.6) |
|  | Strongly Agree | 4 (28.6) |
| **Was the simulator an accurate representation of a thyroid UGFNA experience?** | Strongly Disagree | 0 (0.0) |
|  | Disagree | 2 (14.3) |
|  | Neutral | 4 (28.6) |
|  | Agree | 6 (42.9) |
|  | Strongly Agree | 2 (14.3) |
| **Was the simulator accurate in tactile feedback?** | Strongly Disagree | 0 (0.0) |
|  | Disagree | 2 (14.3) |
|  | Neutral | 6 (42.9) |
|  | Agree | 4 (28.6) |
|  | Strongly Agree | 2 (14.3) |
| **Was the simulator realistic?** | Strongly Disagree | 0 (0.0) |
|  | Disagree | 0 (0.0) |
|  | Neutral | 5 (35.7) |
|  | Agree | 8 (57.1) |
|  | Strongly Agree | 1 (7.1) |
| **Was the simulator valuable?** | Strongly Disagree | 0 (0.0) |
|  | Disagree | 0 (0.0) |
|  | Neutral | 2 (14.3) |
|  | Agree | 4 (28.6) |
|  | Strongly Agree | 8 (57.1) |
| **How many attempts are necessary to become proficient?** | 1-3 | 0 (0.0) |
|  | 4-6 | 11 (78.6) |
|  | 7-9 | 2 (14.3) |
|  | 10+ | 1 (7.1) |
| **Which steps of performing UGFNA did you find benefit from because of the simulator?** † | Long-Axis Technique | 13 (92.9) |
|  | Ultrasound Knobology * | 13 (92.9) |
|  | Short-Axis Technique | 9 (65.3) |

UGFNA: ultrasound-guided fine needle aspiration biopsy

*Ultrasound knobology refers to the correct use of the ultrasound machine and transducer.

† Participants could choose more than one option

Numbers represent frequency (%) for categorical variables.

In the post-study survey outlined here, participants answered a series of questions to determine the face validity and overall perceived value of using the thyroid model. There was a 100% response rate for both the pre-study survey and post-study survey. 10/14 (71.4%) participants agreed/strongly agreed that the sonographic appearance of the thyroid nodules was realistic, and 8/14 (57.1%) of respondents agreed that the simulator was an accurate representation of a thyroid biopsy experience. 85.7% (12/14) of respondents believed that the thyroid model was a valuable tool to augment their learning for UGFNA, and the majority of participants noted that the thyroid simulator was a particularly useful educational tool for learning long-axis biopsy technique (13/14, 92.8%). Simulators previously showed benefit in skill acquisition and retention. Our study has demonstrated the utility, and face validity of the *Blue Phantom Thyroid Model©* with regards to performing UGFNA – ensuring it was an appropriate simulator for the skills studied.
